# A comparative study of neurotoxic potential of synthesized polysaccharide-coated and native ferritin-based magnetic nanoparticles

**DOI:** 10.3325/cmj.2014.55.195

**Published:** 2014-06

**Authors:** Arseniy Borysov, Natalia Krisanova, Olexander Chunihin, Ludmila Ostapchenko, Nataliya Pozdnyakova, Тatiana Borisova

**Affiliations:** 1 Palladin Institute of Biochemistry National Academy of Sciences of Ukraine, Kiev, Ukraine; 2 Educational and Scientific Center “Institute of Biology,” Taras Shevchenko National University in Kiev, Kiev, Ukraine

## Abstract

**Aim:**

To analyze the neurotoxic potential of synthesized magnetite nanoparticles coated by dextran, hydroxyethyl starch, oxidized hydroxyethyl starch, and chitosan, and magnetic nanoparticles combined with ferritin as a native protein.

**Methods:**

The size of nanoparticles was analyzed using photon correlation spectroscopy, their effects on the conductance of planar lipid membrane by planar lipid bilayer technique, membrane potential and acidification of synaptic vesicles by spectrofluorimetry, and glutamate uptake and ambient level of glutamate in isolated rat brain nerve terminals (synaptosomes) by radiolabeled assay.

**Results:**

Uncoated synthesized magnetite nanoparticles and nanoparticles coated by different polysaccharides had no significant effect on synaptic vesicle acidification, the initial velocity of L-[^14^C]glutamate uptake, ambient level of L-[^14^C]glutamate and the potential of the plasma membrane of synaptosomes, and conductance of planar lipid membrane. Native ferritin-based magnetic nanoparticles had no effect on the membrane potential but significantly reduced L-[^14^C]glutamate transport in synaptosomes and acidification of synaptic vesicles.

**Conclusions:**

Our study indicates that synthesized magnetite nanoparticles in contrast to ferritin have no effects on the functional state and glutamate transport of nerve terminals, and so ferritin cannot be used as a prototype, analogue, or model of polysaccharide-coated magnetic nanoparticle in toxicity risk assessment and manipulation of nerve terminals by external magnetic fields. Still, the ability of ferritin to change the functional state of nerve terminals in combination with its magnetic properties suggests its biotechnological potential.

Superparamagnetic iron oxide nanoparticles are a promising candidate for increasing the efficiency of targeted drug delivery and therapy due to external magnetic guidance. Nanomaterials differ from those in bulk forms because they often show unexpected physical and chemical properties. They may produce potential functional and toxicity effects on human nerve cells due to their ability to pass through biological membranes and increase the risk of the development of neurodegenerative diseases (1-3). They can penetrate the blood-brain barrier (3-5) and kill nervous cells in vitro (6-8). Surface modification of iron oxide is a key issue for enhancing its interaction with the cell membrane. By using iron oxide nanoparticles coated by dextran, it was shown that labeled cells could be tracked by magnetic resonance imaging in vivo (9,10). Dextran occupies a special place among polysaccharides because of its wide application. Contrast agents based on dextran-coated iron oxides, eg, Endorem (Guerbet, Roissy, France) and Resovist (Bayer Schering Pharma AG, Berlin-Wedding, Germany), have been commercially available for human use as blood pool agents. Similarly, immortalized cells from the MHP36 hippocampal cell line labeled in vitro with gadolinium rhodamine dextran were tracked in ischemia-damaged rat hippocampus in perfused brains ex vivo (11).

Taking into account that all nanoparticles are more or less toxic and the brain can be a target for their neurotoxic action (3,8,12,13), it is crucial to know their neurotoxic potential. Estimation of neurotoxic risks of nanoparticles can be assessed at various levels of nervous system organization. This research was conducted at the neurochemical level according to the Guidelines for Neurotoxicity Risk Assessment of US Environmental Protection Agency (14), assessing the uptake and release of the neurotransmitters in nerve terminals (15,16). It has been suggested that a possible target for nanoparticles, beyond the already established microglial cells, are presynaptic terminals of neurons (12). Presynaptic nerve terminals contain vesicular pool of neurotransmitters that can be released by exocytosis to the synaptic cleft in response to stimulation (17,18). A key excitatory neurotransmitter in the mammalian central nervous system is glutamate, which is implicated in many aspects of normal brain functioning. Abnormal glutamate homeostasis contributes to neuronal dysfunction and is involved in the pathogenesis of major neurological disorders (19,20). Under normal physiological conditions, extracellular glutamate between episodes of exocytotic release is kept at a low level, thereby preventing continual activation of glutamate receptors and protecting neurons from excitotoxic injury. Low extracellular glutamate concentration is maintained through its uptake by high-affinity Na^+^-dependent glutamate transporters located in the plasma membrane of neurons and glial cells.

Prototypic nanoparticles have been shown to be useful for investigation of synaptic mechanisms underlying the development of neurotoxicity (8,12). Ferritin may be considered as a model nanoparticle (8,12) because it is composed of 24 subunits, which form a spherical shell with a large cavity where up to 4500 ions Fe^3+^ can be deposited as compact mineral crystallites resembling ferrihydrite (21-25). Ferritin stores cellular iron in a dynamic manner allowing the release of the metal on demand (24). Its cores exhibit superparamagnetic properties, which are inherent to magnetic nanoparticles, and vary in diameter from 3.5 nm to 7.5 nm in different tissues (26,27). This protein can penetrate blood-brain barrier (28) and be transported in different cells using clathrin-mediated endocytosis, similarly to many artificial nanoparticles that use the same mechanism (8,29,30).

Recently, there has started an examination of ferritin from the biotechnological point of view. The hypothesis was that ferritin might be considered a good tool and prototypical nanoparticle for investigation of possible toxic properties of metal nanoparticles coated by dextran/polymer shells and possible causes of neurodegeneration associated with exposure to nanoparticles (8,12). Ferritin has been suggested as a label for high-gradient magnetic separation (31) and magnetic force microscopy imaging (32). Recently, it has been shown that the avascular microscopic breast and brain tumors could be noninvasively detected by designing nanoparticles that contained human ferritin as molecular probes for near-infrared fluorescence and magnetic resonance imaging (33).

This research was focused on two aspects – the first was the assessment of neurotoxic potential of synthesized nanoparticles of magnetite (MNP) coated by dextran, hydroxyethyl starch, oxidized hydroxyethyl starch, chitosan as well as uncoated nanoparticles, studying their effects on: 1) the uptake of L-[^14^C]glutamate by rat brain nerve terminals via specific high-affinity Na^+^-dependent plasma membrane transporters; 2) the ambient level of L-[^14^C]glutamate in nerve terminals; 3) the membrane potential (Em) of the plasma membrane of nerve terminals using potential-sensitive fluorescent dye Rhodamine 6G; 4) transmembrane current across the planar lipid membrane using planar lipid bilayer technique; 5) acidification of synaptic vesicles in nerve terminals using pH-sensitive fluorescent dye acridine orange. The second aspect was a comparative analysis of neurotoxic potential of these synthesized polysaccharide-coated nanoparticles and ferritin, which could bring new insight into a possible usage of ferritin as an analogue of polymer-coated magnetic nanoparticle in toxicity risk assessment.

## Materials and methods

### Materials

Ethylene glycol tetraacetic acid (EGTA), 4-(2-hydroxyethyl)-1-piperazineethanesulfonic acid (HEPES), glucose, sucrose, Ficoll 400, and analytical grade salts were purchased from Sigma-Aldrich (St. Louis, MO, USA). Acridine orange and Rhodamine 6G were obtained from Molecular Probes (Eugene, OR, USA) and L-[^14^C]glutamate and aqueous counting scintillant (ACS) from Amersham (Little Chalfont, UK).

Wistar male rats, 100-120 g body weight, were obtained from the vivarium of MD Strazhesko Institute of Cardiology, Medical Academy of Sciences of Ukraine. Animals were kept in animal facilities of the Palladin Institute of Biochemistry in accordance with the European guidelines and international laws and policies (34). They were housed in a quiet, temperature-controlled room (22-23°C) and were given water and dry food pellets *ad libitum*. Rats were decapitated and the brain was removed. Experimental protocols were approved by the Animal Care and Use Committee of the Palladin Institute of Biochemistry (Protocol from September 19, 2012).

### Synthesis of nanoparticles

MNP coated by dextran, hydroxyethyl starch, oxidized hydroxyethyl starch, chitosan as well as uncoated nanoparticles were synthesized at Semenenko Institute of Geochemistry, Mineralogy and Ore Formation, National Academy of Sciences of Ukraine in 2011. Fe_3_O_4_ nanoparticles were obtained by hydrothermal precipitation of Fe^2+^ in aqueous medium in the presence of a weak oxidant in an oxygen-free atmosphere and coated by polysaccharides as described by Mykhaylyk et al (35).

### Analysis of synthesized nanoparticles by dynamic light scattering

The MNP size was measured by dynamic light scattering with a laser correlation spectrometer ZetaSizer-3 (Malvern Instruments, Worcestershire, UK), equipped with He-Ne laser LGN-111 (*P* = 25 mW, λ = 633 nm). The range of measurements is from 1 nm to 50 µm. Suspension (1 mL) was placed in a cylindrical quartz cuvette, 10 mm in diameter, which was set into the laser correlation spectrometer. Registration and statistical processing of laser scattering of suspension of nanoparticles was performed repeatedly for 120 seconds at +22°С with scattering angle of 90°. The results were processed using computer software service PCS-Size mode v. 1.61 (Malvern Instruments). Laser correlation spectrometer was equipped with multi computing correlator type 7032ce (Malvern Instruments).

**Figure 1:**
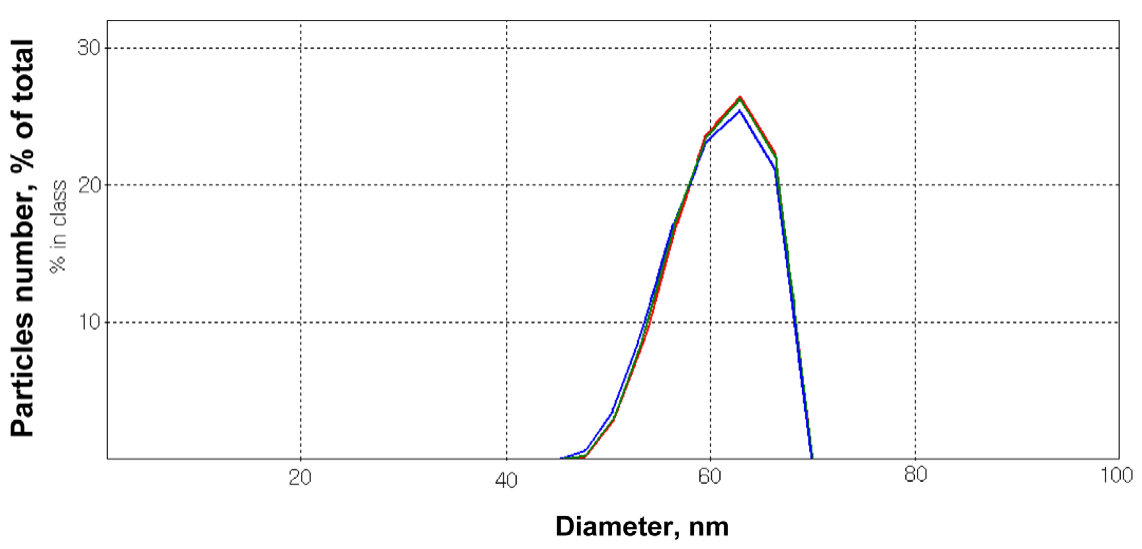
Dynamic light scattering histogram: assessment of the size of magnetite nanoparticles (5 mg/mL) coated by dextran in the standard salt solution. The measurements were performed in 2 minutes.

Surface modification of iron oxide by biocompatible polymers attached to surface of the nanoparticles prevented their agglomeration (36). MNP coated by dextran were assessed using dynamic light scattering (Figure 1). The measurements were performed in a standard salt solution, which contained (in mM) NaCl 126; KCl 5; MgCl_2_ 2.0; NaH_2_PO_4_ 1.0; HEPES 20, pH 7.4; and D-glucose 10. It was demonstrated that suspension of MNP coated by this polysaccharide (5 mg/mL) was stable and the average size of MNP varied between 50-70 nm. The results of dynamic light scattering analysis of MNP coated by hydroxyethyl starch, oxidized hydroxyethyl starch, and chitosan practically did not differ from those of MNP coated by dextran and thus were not shown.

### Isolation of rat brain nerve terminals (synaptosomes) and their interaction with nanoparticles

Cerebral hemispheres of decapitated animals were rapidly removed and homogenized in ice-cold 0.32 M sucrose, 5 mM HEPES-NaOH, pH 7.4, and 0.2 mM EDTA. The synaptosomes were prepared by differential and Ficoll-400 density gradient centrifugation of rat brain homogenate according to the method of Cotman (37) with slight modifications (38-40). All manipulations were performed at 4°C. The synaptosomal suspensions were used in experiments during 2-4 hours after isolation. The standard salt solution was oxygenated. Ca^2+^-supplemented medium contained 2 mM CaCl_2_, Ca^2+^-free-1mM EGTA. Protein concentration was measured as described by Larson et al (41).

Recently, we have evaluated the average size of synaptosomes using dynamic light scattering that was equal to 3.24 ± 0.10 μm (16). In this study, interaction of synthesized polysaccharide-covered MNP and synaptosomes was analyzed. Using dynamic light scattering, it was demonstrated that the average size of synaptosomes (protein concentration of 0.5 mg/mL) was increased by ~ 20% in the presence of MNP coated by different polysaccharides (5 mg/mL).

### L-[^14^C]glutamate uptake by synaptosomes

Synaptosomal suspension (125 μL; 0.2 mg of protein/mL) was pre-incubated in standard salt solution at 37°C for 8 minutes, then nanoparticles were added to the synaptosomal suspension and incubated for 10 minutes. Uptake was initiated by the addition of 10 µM L-glutamate supplemented with 420 nM L-[^14^C]glutamate (0.1 µCi/mL), incubated at 37°C during different time intervals (1, 2, 10 minutes), and then rapidly sedimented using a microcentrifuge (20 seconds at 10 000 g). L-[^14^C]glutamate uptake was determined as a decrease in radioactivity in aliquots of the supernatant (100 μL) and an increase in radioactivity of sodium dodecyl sulfate-treated pellet, and was measured by liquid scintillation counting with ACS scintillation cocktail (1.5 mL).

In the analysis of the functional state of synaptosomes, we applied nanoparticles in a concentration of 2 mg/mL, which was much higher than used in toxicity assessment experiments for iron oxide or titanium oxide nanoparticles (42,43).

### L-[^14^C]glutamate release from synaptosomes

Synaptosomes were diluted in standard salt solution to reach the concentration of 2 mg of protein/mL and after pre-incubation at 37°C for 10 minutes they were loaded with L-[^14^C]glutamate (1 nmol/mg of protein, 238 mCi/mmol) in Ca^2+^-supplemented oxygenated standard salt solution at 37°C for 10 minutes. After loading, the suspension was washed with 10 volumes of ice-cold oxygenated standard salt solution; the pellet was resuspended in a solution to a final concentration of 1 mg protein/mL and immediately used for release experiments. Release of L-[^14^C]glutamate from the synaptosomes was performed in Ca^2+^-free incubation medium according to the following method: synaptosomal suspension (125 μL; 0.5 mg of protein/mL) was pre-incubated for 10 minutes, then MNP (2 mg/mL) were added at 37°C and incubated for 5 minutes and rapidly sedimented using a microcentrifuge (20 seconds at 10 000 g). Release was measured in the aliquots of the supernatants (100 μL) by liquid scintillation counting with scintillation cocktail ACS (1.5 mL). Release of the neurotransmitter from synaptosomes incubated in Ca^2+^-free media without stimulating agents was used for the assay of unstimulated (tonic) release.

### Measurement of synaptosomal plasma membrane potential (E_m_)

Membrane potential was measured using Rhodamine 6G (0.5 µM), which bound with the plasma membrane. Measurements were carried using a Hitachi MPF-4 (Tokyo, Japan) spectrofluorimeter at 528 nm (excitation) and 551 nm (emission) wavelengths (slit bands 5 nm each) (44). The suspension of synaptosomes (0.2 mg/mL of final protein concentration) preincubated at 37°C for 10 minutes was added to stirred thermostated cuvette. To estimate the changes in the plasma membrane potential, the ratio (F) as index of membrane potential was calculated according to Eq 1:

F = F_t_ /F_0_ (1)

where F_0_ and F_t_ are fluorescence intensities of a fluorescent dye in the absence and presence of the synaptosomes, respectively. F_0_ was calculated by extrapolation of exponential decay function to *t* = 0. MNP or ferritin were added at the steady state level of the fluorescent signal.

### Measurements of acidification of synaptosomes

Acridine orange, which is selectively accumulated by the acidic compartments of the synaptosomes (synaptic vesicles), was used for monitoring synaptic vesicle acidification (45,46). Fluorescence changes were measured using a Hitachi MPF-4 spectrofluorimeter at excitation and emission wavelengths of 490 and 530 nm, respectively (slit bands 5 nm each). The reaction was started by the addition of acridine orange (final concentration 5 μM) to synaptosomal suspension (0.2 mg/mL of final protein concentration) preincubated in a stirred thermostated cuvette at 30°C for 10 minutes. The equilibrium level of dye fluorescence was achieved after 3 minutes. Fluorescence (F) was determined according to Eq. 1. MNP or ferritin were added at the steady state level of the fluorescent signal.

### Analysis of the effect of synthesized nanoparticles on the conductance of planar lipid membrane

Planar lipid membrane (0.6 mm in diameter) was formed using the solution of phosphatidylcholine and cholesterol (2:1) in n-heptane, total amount of lipids consisted of 20 mg/mL. Conductance of planar lipid membrane was assessed in a solution consisting of 100 mМ KCl and 10 mM Tris-HCl pH 7.4, which was placed to the both cis- and trans-side of the membrane. Membrane potential was +100 mV. Experiments were carried out at room temperature (22-24°C) in a stirred cuvette. Final concentration of MNP was 0.1 mg/mL.

### Statistical analysis

Results are expressed as means ± standard error of the mean of n independent experiments. Difference between two groups was determined by two-tailed *t*-test. Differences were considered significant at *Р*≤0.05.

## Results

### The presence of synthesized nanoparticles of magnetite did not change membrane potential of nerve terminals

**Figure 2:**
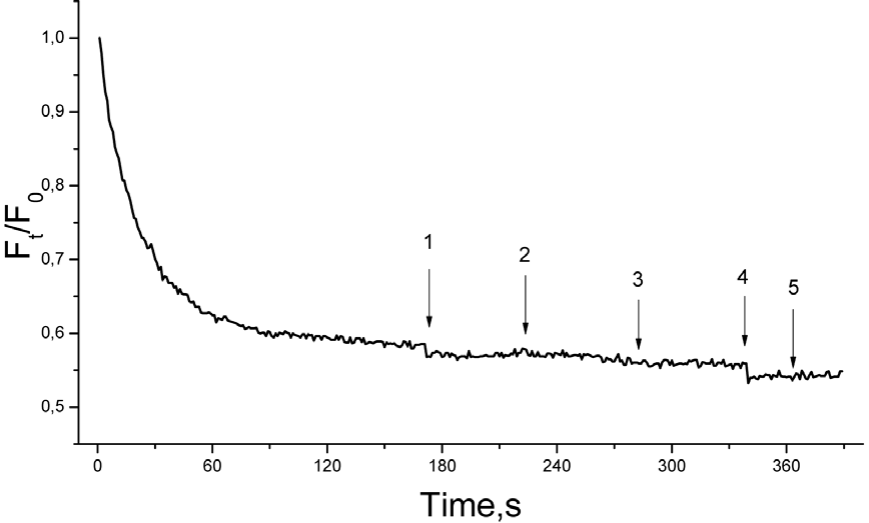
Membrane potential of the synaptosomes after the addition of synthesized magnetite nanoparticles (MNP). The suspension of the synaptosomes was equilibrated with potential-sensitive dye Rhodamine 6G (0.5 µM); when the steady level of the dye fluorescence had been reached, MNP (2 mg/mL) were added (arrows: 1 – uncoated MNP; 2 – MNP coated by dextran; 3 – coated by hydroxyethyl starch; 4 – coated by oxidized hydroxyethyl starch; and 5 – coated by chitosan). Trace represents four experiments performed with different preparations.

Glutamate transporters use Na^+^/K^+^ electrochemical gradients across the plasma membrane as a driving force, and so their functioning depends on the membrane potential (Em). The addition of synaptosomal suspension to the medium containing Rhodamine 6G was accompanied by a partial decrease in fluorescence due to binding of the dye to the plasma membrane (Figure 2). F_st_, the membrane potential index at the steady state level, was achieved for 3 minutes. Synthesized MNP did not influence the fluorescence signal of Rhodamine 6G (Figure 2), reflecting the absence of depolarization of the plasma membrane of nerve terminals.

### The presence of synthesized nanoparticles of magnetite did not change conductance of planar lipid membrane

**Figure 3:**
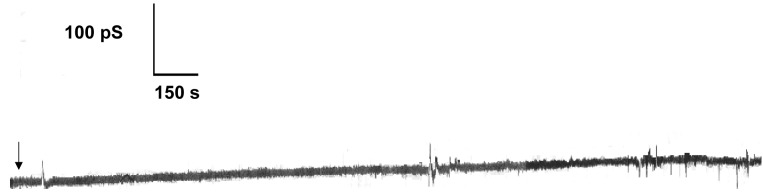
Lack of the influence of synthesized magnetite nanoparticles (MNP) coated by dextran on the conductance of bilayer lipid membrane. MNP (arrow) were added from the cis-side of membrane at a final concentration of 0.1 mg/mL.

The data on the membrane potential of synaptosomes was confirmed by experiments using planar lipid bilayer conductance in the presence of synthesized MNP coated by dextran. After the addition of MNP (0.1 mg/mL) to cis-side of planar lipid membrane, the current across the membrane did not change considerably (Figure 3). These data are in agreement with the results of the assessment of the membrane potential in the presence of synthesized MNP. Uncoated synthesized MNP and MNP coated by hydroxyethyl starch, oxidized hydroxyethyl starch, and chitosan also did not influence the conductance of planar lipid membrane (data not shown).

### The presence of synthesized nanoparticles of magnetite did not change acidification of synaptic vesicles

**Figure 4:**
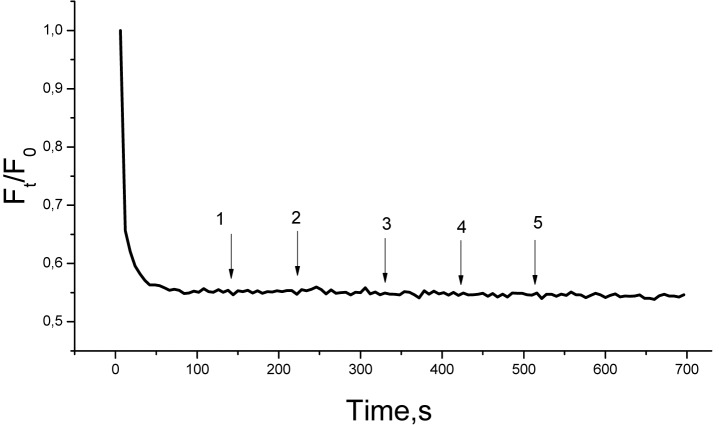
Acidification of synaptosomes in the presence of synthesized magnetite nanoparticles (MNP). The synaptosomes were equilibrated with acridine orange (5 µM); when the steady level of the dye fluorescence had been reached, MNP (2 mg/mL) were added (arrows: 1 – uncoated MNP; 2 – MNP coated by dextran; 3 – coated by hydroxyethyl starch; 4 – coated by oxidized hydroxyethyl starch; and 5 – coated by chitosan). Trace represents four experiments performed with different preparations.

After reaching the cytosol, the amino acid neurotransmitters are accumulated in synaptic vesicles by specific vesicular transporters, which use the proton electrochemical gradient as a driving force. Using acridine orange, synaptic vesicle acidification was measured as an important component of electrochemical proton gradient (Δμ_H_^+^). The addition of acridine orange to synaptosomes was accompanied by partial quenching of fluorescence signal due to dye accumulation in synaptic vesicles. After loading with acridine orange, synthesized MNP were added to synaptosomes at a concentration of 2 mg/mL. The addition of these nanoparticles did not influence the fluorescence of acridine orange, indicating that synaptic vesicles retained appropriate proton gradient in the presence of MNP (Figure 4).

### The presence of synthesized nanoparticles of magnetite did not change glutamate uptake and the extracellular level of glutamate in nerve terminals

**Figure 5:**
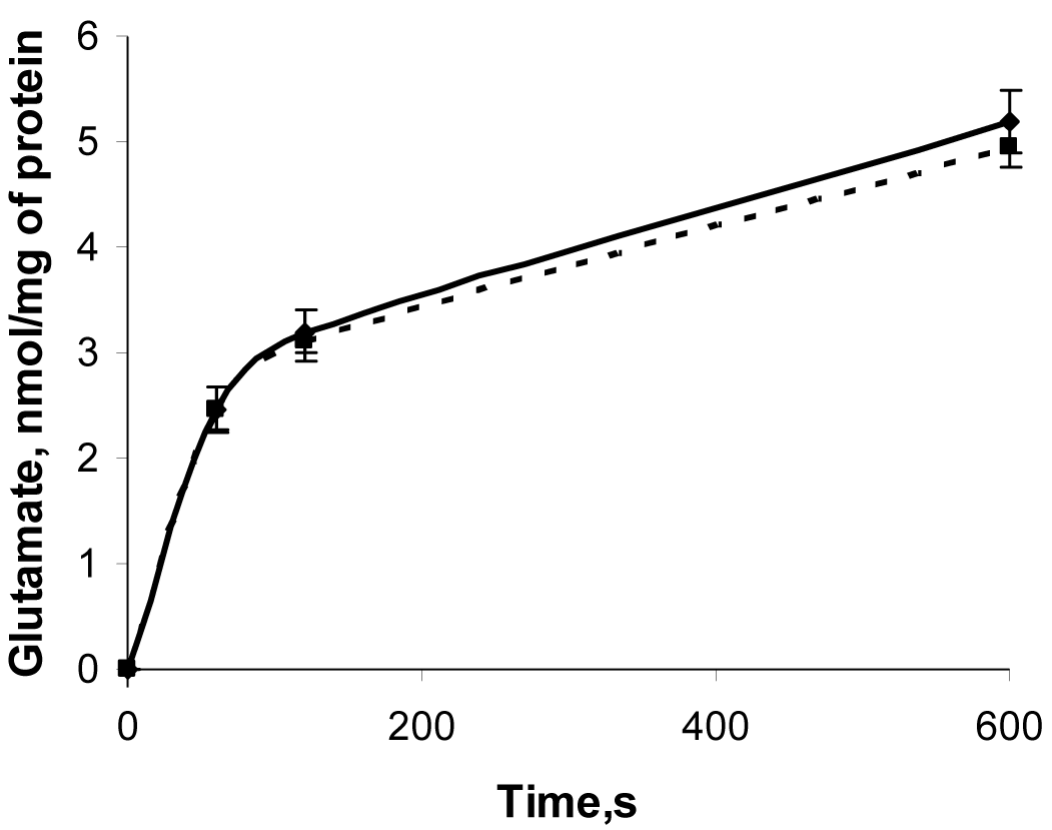
Time course of uptake of L-[^14^C]glutamate by control synaptosomes (solid line); synaptosomes in presence of dextran-coated magnetite nanoparticles (MNP) (2 mg/mL) (dashed line). Uptake was initiated by the addition of L-[^14^C]glutamate to synaptosomes, after incubation the samples were rapidly sedimented and radioactivity was determined as described in Materials and methods. Data are presented as mean ± standard error of the mean of three independent experiments.

Incubation of synthesized MNP coated by dextran with synaptosomes for 10 minutes did not cause significant changes in the initial velocity of high affinity Na^+^-dependent L-[^14^C]glutamate uptake and accumulation of L-[^14^C]glutamate (Figure 5). The initial velocity of L-[^14^C]glutamate uptake by nerve terminals was equal to 2.5 ± 0.2 nmol min^-1^ mg^-1^ protein in the control experiments and 2.45 ± 0.2 nmol min^-1^ mg^-1^ protein in the presence of dextran-coated MNP. Uncoated, hydroxyethyl starch-, oxidized hydroxyethyl starch-, and chitosan-coated MNP also did not influence the initial velocity of L-[^14^C]glutamate uptake and accumulation of L-[^14^C]glutamate by synaptosomes (data not shown).

In this set of the experiments, the extracellular level and tonic release of glutamate from nerve terminals in the presence of uncoated, and dextran-, hydroxyethyl starch-, oxidized hydroxyethyl starch-,and chitosan-coated MNP were analyzed. After 6 minutes of incubation, the extracellular level of L-[^14^C]glutamate in synaptosomal suspension consisted of 204 ± 3 pmol of L-[^14^C]glutamate/mg of protein in the control experiment and insignificantly varied within the range of 5% in the presence of all types of synthesized MNP. Therefore, we did not find significant changes (*P* ≤ 0.10) in glutamate uptake and the extracellular level of glutamate in nerve terminals in the presence of synthesized MNP.

### Comparison of the neurotoxic potential of synthesized polysaccharide-coated nanoparticles of magnetite and native ferritin-based magnetic nanoparticles

**Figure 6:**
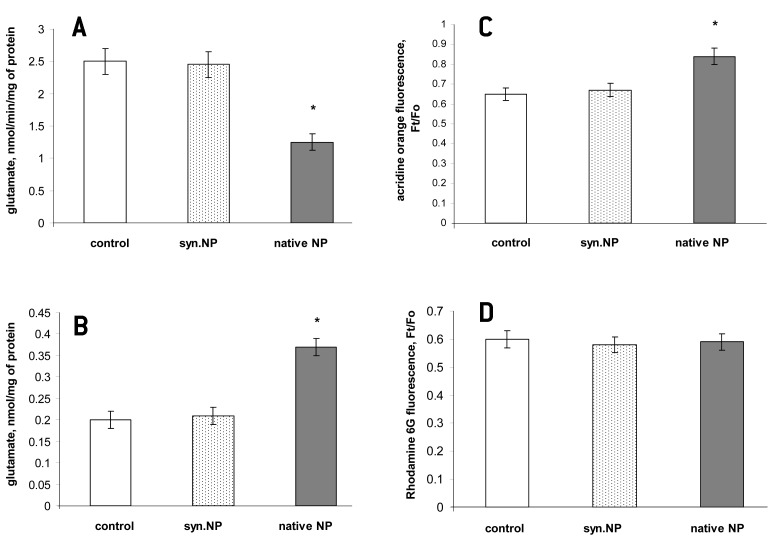
Comparison of the effects of synthesized magnetite nanoparticles (MNP) coated by dextran (2 mg/mL) (synthesized nanoparticle) and ferritin (100 μg/mL) (native nanoparticle) on the initial velocity of L-[^14^C]glutamate uptake by synaptosomes (**A**), the ambient level of L-[^14^C]glutamate at 5 minutes time point (**B**), synaptic vesicle acidification (**C**), and the membrane potential (**D**). Data are presented as mean ± standard error of the mean of three independent experiments. *Р≤0.05 as compared to control.

We correlated the effects of synthesized polysaccharide-coated MNP (dextran-coated nanoparticles at a concentration of 2 mg/mL were used as an example) and ferritin (100 μg/mL) on the initial velocity of L-[^14^C]glutamate uptake by synaptosomes (Figure 6A), the ambient level of L-[^14^C]glutamate (Figure 6B), synaptic vesicle acidification (Figure 6C), and the membrane potential (Figure 6D). Uncoated, hydroxyethyl starch-, oxidized hydroxyethyl starch-, and chitosan-coated MNP demonstrated similar effects as dextran-coated MNP. It was found that except the membrane potential (Figure 6D), all other key characteristics of glutamatergic neurotransmission were changed as a result of ferritin application, ie, the initial velocity of L-[^14^C]glutamate uptake was decreased (Figure 6A), the extracellular level of L-[^14^C]glutamate was increased in nerve terminals (Figure 6B), and the proton electrochemical gradient of synaptic vesicles was reduced, which indicates an increase in fluorescence of acridine orange (Figure 6C). Therefore, synthesized polysaccharide-coated MNP and native ferritin-based magnetic nanoparticles differently influenced the functional state of nerve terminals.

## Discussion

Little is known about the involvement of excitotoxicity in nanoparticle-induced neuronal death (8,12). In this study, we assessed neurotoxic/excitotoxic potential of synthesized MNP coated by dextran, hydroxyethyl starch, oxidized hydroxyethyl starch, chitosan as well as uncoated MNP. It should be noted that dextran-coated iron oxides are widely used in biomedicine as supermagnetic agents for magnetic resonance imaging, for labeling of different cells and tissues, and transfection (9-11). Neurotoxicity risk assessment of synthesized and native nanoparticles was performed using isolated rat brain nerve terminals. Synaptosomes retain all characteristics of intact nerve terminals, that is, the ability to maintain membrane potential, accomplish glutamate uptake, exocytosis, etc. Synaptosomes are one of the best systems to explore the relationship between the structure of proteins, their biochemical and cell-biological properties, and physiological role (47). We demonstrated that synthesized polysaccharide-coated MNP did not influence the initial velocity of L-[^14^C]glutamate uptake and the ambient level of L-[^14^C]glutamate in nerve terminals. It should be noted that a proper level of ambient glutamate is very important for synaptic transmission, whereas an increase in this level causes neurotoxicity. The ambient level of the neurotransmitter is determined by a balance between its uptake and tonic release from nerve terminals. Origin of tonic release is not completely identified. It is suggested that glutamate enriches the extracellular space by spontaneous exocytosis, swelling-activated anion channels, cysteine-glutamate exchange, trans-membrane diffusion, and volume-sensitive Cl^-^ channels (48). Acidification of synaptic vesicles, the membrane potential of nerve terminals and transmembrane current across the planar lipid membrane were not changed as a result of the application of these nanoparticles. Therefore, it was concluded from the in vitro study that synthesized polysaccharide-coated MNP did not change the functional state of nerve terminals.

Nanoparticles can be used in targeted drug delivery, as labels in different diagnostics methods or scaffolding materials in neuronal regeneration (8,12,13) In experiments on mice, silica-coated cobalt ferrite nanoparticles were found in the brain after intravenous injection (49). Nanoparticles of TiO_2_ transferred from pregnant mice to their offspring can damage the genital and cranial nerve systems (50). According to one explanation, nanoparticles can be transported into the cells through endocytosis (51,52) and according to another by diffusion or adhesive interactions (53). Olfactory mucosa and the olfactory bulb of rat brain could provide a portal for entry of intranasally instilled nanoparticles into the central nervous system circumventing the blood-brain barrier (54).

Recently, it has been hypothesized that ferritin could be a promising tool for assessment of possible toxic properties of metal nanoparticles coated by dextran/polymer and a good prototypical nanoparticle for investigation of probable causes of neurodegeneration associated with exposure to nanoparticles (8,12). Ferritin application can be supported by several facts: 1) it consists of a hydrous ferric oxide phosphate particle coated by protein shell (21-25); 2) its cores exhibit superparamagnetic properties, which are inherent to magnetic nanoparticles (26); 3) ferritin can cross the blood-brain barrier like many nanoparticles (29); 4) ferritin can be transported in different cells using clathrin-mediated endocytosis similarly to many synthesized nanoparticles (8,29,30). In this study, we comparatively analyzed the neurotoxic potential of synthesized and native nanoparticles and demonstrated that in contrast to synthesized polysaccharide-coated MNP, ferritin significantly decreased the initial velocity of L-[^14^C]glutamate uptake by nerve terminals, and as a consequence, increased the ambient level of L-[^14^C]glutamate in nerve terminals. Ferritin reduced the proton electrochemical gradient of synaptic vesicles, whereas synthesized MNP did not affect this parameter. It should be noted that ferritin-induced decrease in acidification of synaptic vesicles may be one of the causes of weak glutamate uptake and enhanced extracellular level of glutamate in nerve terminals. We suppose that the observed effects of ferritin were not associated with the influence of exogenous iron ions. This is because release of iron ions from ferritin occurs mainly through lysosomal proteolysis and requires lysosomal activity (55). Therefore, synthesized and native nanopatrticles have diverse effects on the functional state of nerve terminals, whereas their similar feature was the absence of influence on the potential of the plasma membrane of nerve terminals.

The main limitation of this study was the difference in the size of synthesized polysaccharide-coated MNP, which varied between 50-70 nm in a standard salt solution, and ferritin molecule, whose core diameter was ~ 7.5 nm (26,27). This difference in inorganic core may be crucial for the process of interaction of synthesized and native nanoparticles with nerve terminals, which results in diverse functional effects on glutamate transport. However, we also used uncoated MNP subjected to sonication, the average diameter of which was ~ 10 nm (data not shown). The effects of these uncoated and polysaccharide-coated MNP on the key characteristics of glutamatergic neurotransmission were similar despite the difference in their size. Therefore, it can be suggested that the difference in the size of synthesized and native nanoparticles was not a cause of the observed changes in the key characteristics of glutamatergic neurotransmission.

In conclusion, synthesized polysaccharide-coated MNP did not significantly influence the functional state of nerve terminals and key characteristics of glutamatergic transmission, whereas native ferritin-based magnetic nanoparticles considerably affected the proton gradient and glutamate transport in nerve terminals. These data indicate that ferritin could be used neither as a model/analogue of polymer-coated magnetic nanoparticles in the assessment of their toxic properties and health risks during diagnostic labeling and manipulation by external magnetic fields, nor as a prototypical nanoparticle for investigation of possible causes of neurodegeneration associated with exposure to nanoparticles. Still, it could have a biotechnological potential due to its magnetic properties in combination with the ability to change the functional state of nerve terminals.
